# Supplementary material for: Innovative Collagen Based Biopolymers Tested as Fertilizers for Poor Soils Amendment
Source: Polymers (Basel). 2023 Apr 27;15(9):2085. doi: 10.3390/polym15092085 (PMC10181486; doi:10.3390/polym15092085)
Supplement: Supplementary file 1 [file polymers-15-02085-s001.zip › polymers-2280240-supplementary.pdf]

polymers MDPI

Table S1. Absolute locations of sampling points for Greek and Romanian soils

Geographical Location	Country	Code	GPS Coordinates	
			North	East
**Neochori Messolonghiou**	Greece	S1	38°24'39''	21°16'29''
**Kernitsa Achaias**	Greece	S2	38°07'24''	22°10'53''
**University of Patras**	Greece	S3	38°17'24''	21°47'13''
**Aldeni, Buzau County**	Romania	S4	45°19'08''	26°45'24''

Table S2. Soil texture

Soil sample	Particle size (mm) distribution (%)											Textural class Symbol
	Coarse sand				Fine sand				Silt	Clay		
	**2-0.2**	**2-1**	**1-0.5**	**0.5-0.2**	**0.2-0.02**	**0.2-0.1**	**0.1-0.05**	**0.05-0.02**	**0.02**	**0.002**	**0.01**	
**S1**	**28.1**	10.5	6.6	11.0	**29.3**	6.7	2.5	20.1	**19.6**	**23.0**	36.8	L
**S2**	**14.4**	4.1	4.0	6.3	**22.1**	5.5	2.7	13.9	**19.5**	**44.0**	57.9	CL
**S3**	**6.4**	1.4	1.1	3.9	**7.1**	3.0	1.6	2.5	**46.8**	**39.7**	57.7	SiCL
**S4**	**35.1**	12.5	4.9	14.7	**36.7**	25.5	10.3	0.9	**1.2**	**27.0**	36.0	SCL
